# Advancing Sustainability and Performance with Crushed Bottom Ash as Filler in Polymer-Modified Asphalt Concrete Mixtures

**DOI:** 10.3390/polym16121683

**Published:** 2024-06-13

**Authors:** Yeong-Min Kim, Kyungnam Kim, Tri Ho Minh Le

**Affiliations:** 1 Department of Highway & Transportation Research, Korea Institute of Civil Engineering and Building Technology, 283 Goyangdae-Ro, Ilsanseo-Gu, Goyang-si 10223, Gyeonggi-do, Republic of Korea; choozang@kict.re.kr; 2 Korea Expressway Corporation Research Institute, Pavement Research Division, Dongtansunhwan-daero 17-gil, Hwaseong-si 18489, Gyeonggi-do, Republic of Korea; 3 Faculty of Civil Engineering, Nguyen Tat Thanh University, 300A Nguyen Tat Thanh Street, District 4, Ho Chi Minh City 70000, Vietnam

**Keywords:** sustainable pavement, recycled materials, bottom ash filler, polymer-modified asphalt, crumb rubber, performance evaluation

## Abstract

Amid the growing demand for sustainable pavement solutions and the need to incorporate recycled materials into construction practices, this study explored the viability of using crushed thermal power plant bottom ash as a filler in polymer-modified asphalt concrete mixtures. Conventional lime filler was replaced with bottom ash at varying levels (0%, 25%, 50%, and 75%), and the resulting mixtures were evaluated using several performance tests. The optimal replacement level was determined to be 25%, based on the results of the indirect tensile strength (ITS) test. Comparisons between the control mixture and the 25% bottom ash-modified mixture were conducted using the dynamic modulus test, Cantabro test, Hamburg wheel tracking (HWT) test, and tensile strength ratio (TSR) test. The findings indicate that the 25% bottom ash-modified mixture demonstrated improved performance across multiple parameters. The HWT test showed enhanced rut durability, with a recorded depth of 7.56 mm compared to 8.9 mm for the control mixture. The Cantabro test results revealed lower weight loss percentages for the modified mixture, indicating better abrasion resistance. The dynamic modulus test indicated higher resilience and stiffness in both high- and low-frequency stages. The TSR test highlighted improved moisture resistance, with higher TSR values after 10 wet-drying cycles. These improvements are attributed to the fine particle size and beneficial chemical composition of bottom ash, which enhance the asphalt mixture’s density, binder-aggregate adhesion, and overall durability. The results suggest that incorporating 25% crushed bottom ash as a filler in polymer-modified asphalt concrete mixtures is a viable and sustainable approach to improving pavement performance and longevity.

## 1. Introduction

The ever-increasing demand for sustainable construction materials has driven significant research into alternative materials that can enhance the performance of asphalt concrete mixtures while mitigating environmental impacts [1]. Among the various types of asphalt concrete, polymer-modified asphalt (PMA) has gained substantial attention due to its superior performance characteristics, including increased stiffness, reduced deformation, and enhanced durability [2]. These benefits are particularly pronounced in styrene-butadiene-styrene (SBS) modified binders, which have been widely adopted in road construction due to their ability to withstand a range of climatic conditions and heavy traffic loads [3].

SBS-modified asphalt mixtures highlight their superior performance characteristics compared to conventional asphalt mixtures [4,5]. SBS modification enhances the rheological properties of asphalt binders, resulting in increased stiffness and elasticity, which are crucial for maintaining pavement performance under varying environmental conditions and traffic loads [6,7]. Studies have shown that SBS-modified mixtures possess improved fatigue resistance due to the polymer’s ability to enhance flexibility and tensile strength, thereby reducing crack initiation and propagation [8,9]. Furthermore, SBS modification greatly increases the binder’s high-temperature efficiency, which is crucial for preventing rut development under heavy traffic, hence improving rutting resistance [10,11]. SBS-modified mixtures also exhibit enhanced resistance to thermal cracking, as the polymer provides greater elasticity, allowing the binder to better accommodate thermal stresses [12]. Furthermore, SBS-modified asphalt demonstrates better long-term aging performance, maintaining flexibility and adhesion properties over time, which is vital for pavement durability [13]. Related reports note that SBS-modified mixtures have lower moisture susceptibility due to improved binder-aggregate adhesion, reducing the potential for stripping and moisture-induced damage [2]. Collectively, these studies underscore the significant advantages of SBS-modified asphalt mixtures, making them a valuable choice for designing high-performance pavements capable of meeting modern infrastructure demands.

Despite the advantages of SBS-modified asphalt, the search for further performance improvements and sustainability has led to the exploration of various additives and fillers. One promising avenue is the incorporation of recycled crumb rubber from tires [14], which not only improves the performance of HMA mixtures but also addresses the growing concern of tire waste management. Crumb rubber-modified asphalt has been shown to enhance flexibility, resistance to thermal cracking, and overall longevity of pavements [15,16].

Recent studies have shown that using byproducts and waste materials in hot mix asphalt (HMA) can significantly enhance its sustainability and performance [17,18]. Research has demonstrated that bottom ash can improve moisture resistance and reduce environmental impacts [19,20]. Other studies have found that coal bottom ash and carbonized rice husk can be effectively used in HMA [21,22]. Low-grade magnesium carbonate by-products and palm oil by-products have also been explored for their beneficial properties in asphalt mixtures [22]. Additionally, waste engine oil and waste plastic aggregates, along with additives like magnesium, have been evaluated for their positive impact on eco-friendly asphalt mixtures [23,24]. The performance of recycled asphalt mixtures has been improved using advanced separation technologies [25]. Overall, these findings highlight the practical benefits of incorporating waste materials into HMA, contributing to more sustainable construction practices [26].

In parallel, the utilization of industrial by-products as fillers in asphalt mixtures has garnered interest due to their potential to reduce environmental impact and material costs [27]. Thermal power plant bottom ash is one such by-product that has shown promise as a filler material [26]. Bottom ash, a residue from coal combustion in power plants, is primarily composed of silica, alumina, and other oxides, which can contribute to the mechanical strength and durability of asphalt mixtures [18]. Reducing the amount of natural resources used and diverting large amounts of waste from landfills are achievable by mixing bottom ash into asphalt blends.

Despite the numerous benefits of SBS-modified asphalt mixtures, there are still some limitations and areas that require further research. One significant limitation is the increased cost associated with SBS polymers, which can make large-scale implementation economically challenging. Additionally, the performance of SBS-modified asphalt can vary based on the specific formulation and mixing process, leading to inconsistencies in field applications. There is also a need for more extensive long-term performance data to fully understand the durability of these mixtures under diverse climatic conditions and traffic loads. Research needs include optimizing the mix design to reduce costs, improving the understanding of the interactions between SBS polymers and different types of aggregates, and developing standardized procedures for incorporating SBS into asphalt mixtures to ensure consistent quality. This research addresses these gaps by investigating the feasibility of using crushed thermal power plant bottom ash as a filler in SBS-modified asphalt mixtures containing crumb rubber. This innovative approach not only aims to enhance the performance and sustainability of the asphalt but also provides a cost-effective solution by utilizing industrial by-products. By exploring the optimal replacement levels of conventional lime with bottom ash, our study contributes to the growth of more durable, eco-friendly, and economically viable pavement materials.

**Figure 1:**
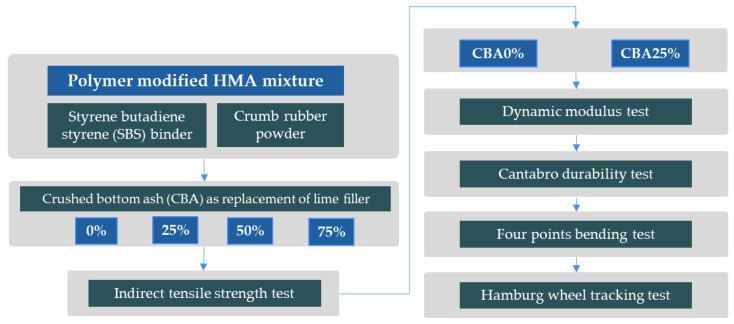
Research flowcharts.

The primary goal of this research was to evaluate the feasibility of using crushed thermal power plant bottom ash as a filler in polymer-modified asphalt concrete mixtures. To accomplish this, this study used the Superpave mix design approach, which aimed for an ideal asphalt binder content. This study involved replacing conventional lime filler with bottom ash at replacement levels of 0%, 25%, 50%, and 75%, to determine the most effective proportion. The asphalt combinations were developed utilizing a mix of SBS-modified binder from asphalt and particular proportions carefully chosen to improve performance characteristics. The research then produced samples for each mix design and subjected them to a series of rigorous tests, including the indirect tensile strength (ITS) test, dynamic modulus test, Cantabro test, Hamburg wheel tracking (HWT) test, and tensile strength ratio (TSR) test. These tests were conducted to evaluate the mechanical properties, durability, moisture susceptibility, and overall performance of the modified asphalt mixtures. Through these comprehensive evaluations, our research aimed to demonstrate the potential benefits and practical applications of using bottom ash as a sustainable alternative filler in asphalt pavements. The research flowchart is presented in the following Figure 1.

## 2. Materials and Methods

### 2.1. Materials

#### 2.1.1. Aggregate

The aggregate utilized in this research had a nominal maximum aggregate size (NMAS) of 10 mm and was specifically designed to meet the parameters for HMA procedures. This material met strict national criteria for aggregate quality and came from a reliable quarry [28]. In compliance with specified aggregate regulations, an extensive assessment of the aggregate’s physical characteristics was carried out. The aggregate was meticulously cleaned and dried before being added to the asphalt concrete compositions. It was heated at 110 °C for 24 h to remove any remaining moisture and contaminants. According to national guidelines, a sieve test was then carried out to guarantee the aggregate’s consistency and appropriate gradation, as shown in Table 1. Certain physical and mechanical characteristics of the aggregate were included in the quality assessment; these are all thoroughly recorded in Table 2.

#### 2.1.2. Characterization of Bottom Ash

The crushed bottom ash used in this study was characterized to understand its properties and justify its effects on the modified asphalt mixtures. The particle size distribution of the bottom ash particles was analyzed using sieve analysis, which revealed a size distribution ranging from 0.1 to 100 μm, with a significant proportion of particles below 50 μm. Table 3 presents the overview of the bottom ash properties used in this research.

The porosity and surface area were measured using a BET (Brunauer-Emmett-Teller) analysis, which found the specific surface area of the bottom ash to be 25 m^2^/g, indicating a high surface area conducive to good binder-aggregate interaction. Additionally, the porosity was measured using mercury intrusion porosimetry, revealing a total porosity of 40%. The density of crushed bottom ash was found to be 2.7 g/cm^3^, higher than conventional lime filler, contributing to the mixture’s overall density and stability.

The chemical composition was analyzed using X-ray fluorescence (XRF), showing the following composition: 50% SiO_2_, 28% Al_2_O_3_, 12% Fe_2_O_3_, 6% CaO, 2% MgO, and 2% SO_3_. These characteristics underscore the suitability of crushed bottom ash as a filler in asphalt mixtures, contributing to enhanced binder-aggregate adhesion and overall mixture performance.

Silicon dioxide and aluminum oxide enhance the hardness and strength of the bottom ash, improving the asphalt’s load-bearing capacity. Iron oxide boosts thermal stability and resistance to high temperatures, essential for varying climates. Calcium oxide improves binder-aggregate adhesion, reducing moisture damage and stripping. Magnesium oxide and sulfur trioxide further enhance the asphalt mixture’s chemical stability and performance.

**Figure 2:**
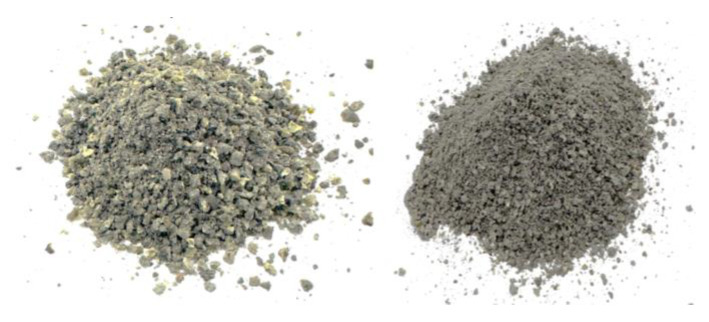
Bottom ash before and after the crushing process.

In this research, the filler ratio was fixed at 1.5% by weight for both lime and crushed bottom ash based on initial trial tests [28]. The additives used in this research, including crushed bottom ash (see Figure 2) and other components, were meticulously selected and characterized to evaluate their suitability for asphalt mixture applications. Conventional lime filler exhibits variable particle sizes, depending on the source and processing methods, while crushed bottom ash presents a more uniform particle size distribution ranging from 0.1 to 100 μm.

Crushing bottom ash is essential to optimize its suitability for incorporation into asphalt mixtures and other construction applications [29]. Reducing bottom ash to finer particle sizes, enhances its compatibility with asphalt binder and aggregates, promoting better homogeneity and cohesion within the mixture. Additionally, crushing facilitates better distribution of bottom ash throughout the asphalt matrix, ensuring uniformity of properties and enhancing overall performance. The process of crushing also helps to mitigate potential issues related to particle size distribution, ensuring that the bottom ash meets specified requirements for the intended application. Overall, crushing bottom ash is a crucial step in unlocking its potential as a sustainable filler material, contributing to the development of eco-friendly and high-performing pavement solutions.

In terms of density, conventional lime filler typically ranges from 2.2 to 2.5 g/cm^3^, whereas crushed bottom ash demonstrates a higher density of 2.7 g/cm^3^. Chemical composition analysis highlights further disparities between the two materials [30]. Conventional lime filler primarily consists of calcium carbonate (CaCO_3_), whereas crushed bottom ash comprises a diverse composition. Its chemical composition includes 50% silicon dioxide (SiO_2_), 28% aluminum oxide (Al_2_O_3_), 12% iron oxide (Fe_2_O_3_), 6% calcium oxide (CaO), 2% magnesium oxide (MgO), and 2% sulfur trioxide (SO_3_). This composition reflects the mineralogical nature of bottom ash, making it a potentially effective filler material in asphalt mixtures [30].

In addition, bottom ash’s absorption properties were evaluated. The crushed bottom ash demonstrated an absorption rate of 0.4%, significantly lower than the 1.3% absorption rate of natural aggregates. This lower absorption rate suggests that crushed bottom ash may offer enhanced resistance to moisture-induced damage in asphalt mixtures, potentially leading to improved durability and performance.

#### 2.1.3. Asphalt Binder Modified with Styrene-Butadiene-Styrene Polymer

A polymer-modified SBS asphalt binder was incorporated into the mixture to improve its rheological properties. This SBS binder, introduced in pellet form during mixing, serves to enhance stiffness and durability, addressing the demands of high-performance asphalt pavements. The quantity of SBS polymer was carefully determined based on expert recommendations and preliminary trials [31] as outlined in Table 4, ensuring optimal performance. Furthermore, crumb rubber powder, detailed in Table 5, was introduced at a rate of 6% to augment the overall performance of the asphalt mixture. This addition followed a meticulous treatment process to eliminate contaminants and enhance the rubber’s compatibility with the binder. The treatment process involved cleaning the crumb rubber to remove any residual contaminants, followed by a cryogenic grinding method to achieve a uniform particle size distribution. The rubber particles were then treated with a chemical activator to improve the bonding with the asphalt binder [32,33]. Additionally, a 1% dosage of epoxy resin was included to bolster the strength and durability of the asphalt. Epoxy resin, renowned for its thermosetting properties, forms a resilient bond with the binder and aggregates, effectively reinforcing the asphalt matrix against various stressors. Integration of the epoxy resin occurred during mixing to facilitate thorough dispersion throughout the mixture. Table 6 provides a detailed summary of the parameters of the modified asphalt binder, which incorporates SBS and various additions.

#### 2.1.4. Mix Design

This study made use of the Superpave mix design technique, which is a thorough and widely acknowledged strategy to improving asphalt binder content and aggregate gradation [41]. This technique takes into account crucial elements such as the amount of traffic, weather circumstances, and pavement specifications. The mix design aims to produce an air void content of 4.5% while maintaining appropriate VMA (voids in mineral aggregate) at 18% and VFA (voids filled with asphalt) at 75% [28]. A 6.5% polymer-modified asphalt binder is a key component of this mix formulation. This binder is specifically designed to utilize crushed bottom ash as a filler ingredient. The incorporation of bottom ash not only maximizes recyclables for the benefit of the environment, but it also strives to improve the asphalt mixture’s structural characteristics. Along with the bottom ash, the mix contained 19.2% fine aggregate and 75.5% coarse aggregate by weight, resulting in a balanced and effective composition.

In the laboratory, the preparation of asphalt mixture involved several essential steps. Initially, the aggregate was heated to a precise temperature of 165 °C using a dryer drum. Following this, the crushed bottom ash filler was added to the heated aggregate and mixed thoroughly for about 2–4 min. It was crucial to maintain the temperature at 155–165 °C throughout the mixing process to ensure proper blending and uniformity of the mixture. After mixing, the mixture was compacted using the Superpave gyration compactor method to achieve the desired volumetric properties and density.

The mixed design criteria, as outlined by the Ministry of Land, Infrastructure, and Transport (MOLIT) 2017 standards [42], specify targets for the MC-1 layer. These targets include 75 gyrations, an air void content ranging from 3 to 6%, a minimum VMA of 13.0%, and a VFA between 70 and 85%. The mixture must also meet several performance benchmarks: a deformation strength of at least 3.2 MPa, a tensile strength ratio of at least 0.8, and an indirect tensile strength (ITS) of at least 0.8 MPa. Since bottom ash is an inert material and does not require curing, the emphasis is on optimizing the blending and compaction process to achieve the desired mechanical properties and performance characteristics of the asphalt mixture. Using bottom ash filler not only enhances sustainability by repurposing waste materials but also potentially improves the mechanical properties and long-term performance of the asphalt mixture, thereby contributing to sustainable infrastructure development.

### 2.2. Testing Methods

#### 2.2.1. Indirect Tensile Strength Test

**Figure 3:**
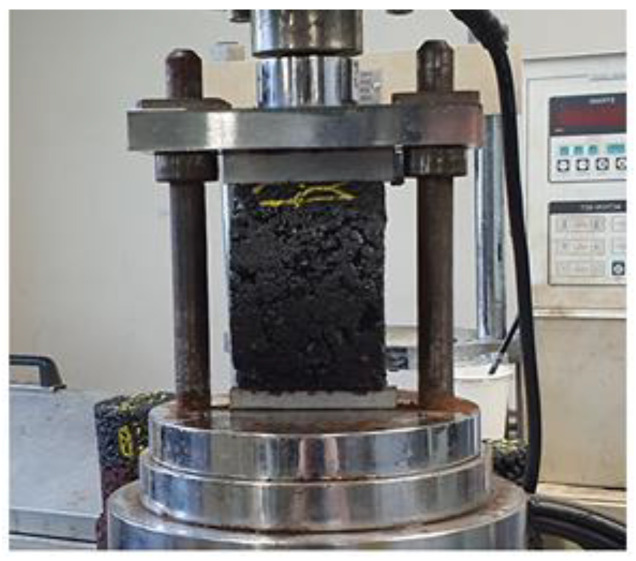
Indirect tensile strength test.

In this study, the ITS experiment was carried out in compliance with the ASTM D6931 guideline to determine the tensile strength of the asphalt samples [43]. The experiment was conducted using a D25 Universal Testing Machine (UTM) with a regulated displacement rate (see Figure 3), manufactured in South Korea. Standard samples for ITS evaluation are 100 mm in diameter and 60 mm in height. Throughout the procedure, the samples were exposed to diametral pressure at a continuous deformation rate until failure.

The ITS was developed to assess the combination’s durability against constant deformation in both wet and dry circumstances. The samples were aged by subjecting them to hot air and temperatures for three hours and then immersing them in 80 °C water for twenty-four hours. For the ITS tests at 60 °C, the samples were preconditioned to the desired temperature by placing them in a T10 temperature-controlled chamber set at 60 °C for two hours prior to testing. The T10 chamber is manufactured in South Korea. This ensured that the entire specimen reached the target temperature uniformly. The ITS apparatus, though typically operating at room temperature, was supplemented with an environmental chamber to maintain the required temperature during the test. The ITS was calculated using the following formula:

(1) $$\mathrm{ITS}=\frac{2P}{\pi tD}$$

where:

- P is the maximum load at failure (N),
- t is the specimen height (mm),
- D is the specimen diameter (mm).

ITS was computed at both 25 °C and 60 °C, in both dry and wet circumstances. The ITS test loading rate of 50 mm/min guaranteed a steady and regulated load application, allowing precise assessment of the asphalt concrete specimens’ tensile strength characteristics.

This method ensured that the ITS values reflected the performance of the asphalt mixtures under different temperature conditions, thereby providing a comprehensive evaluation of their mechanical properties and durability.

#### 2.2.2. Dynamic Modulus Test

**Figure 4:**
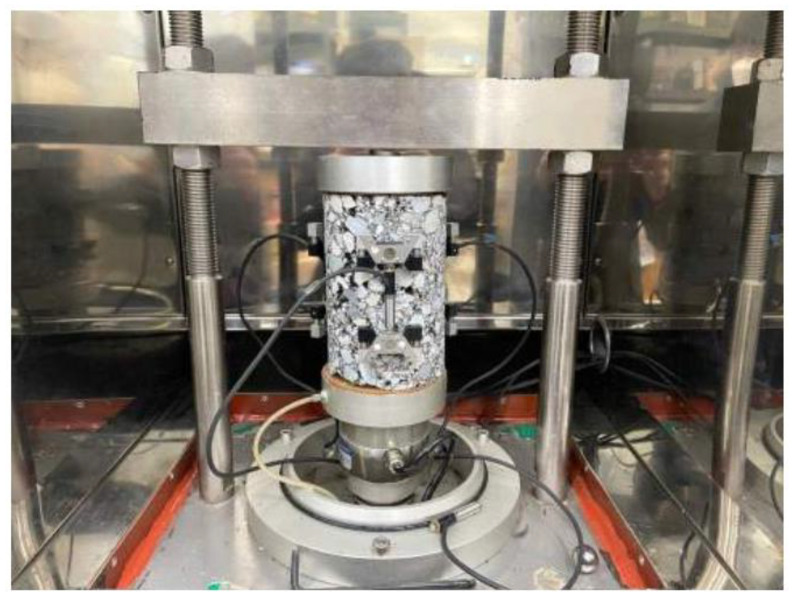
Dynamic modulus test apparatus.

The test evaluates the viscoelastic and stiffness characteristics of asphalt concrete under various loading conditions (see Figure 4). This study employed a Universal Testing Machine (UTM), specifically the DTS-30 model from MATEST (Italy), with a dynamic modulus device on cylindrical specimens (100 mm diameter, 150 mm height) following AASHTO TP 62 [44]. Temperatures ranging from −10 °C to 54 °C and frequencies from 25 Hz to 0.1 Hz were tested. The obtained phase angle (δ) and complex modulus (E*) data were utilized to construct a master curve through time-temperature superposition that illustrated stiffness at various loading rates and temperatures. This experiment verified the increased stiffness and viscoelastic properties of the suggested polymer-modified asphalt concrete combination. The viscosity η of the binder was calculated using the following formulae:

(2) $$\log\left(log\eta \right)=A+VTS\left[\log\left(T_{R}\right)\right]$$

(3) $$log\left(T\right)=c\left(10^{A+VTS\left[\log\left(T_{r}\right)\right]}-10^{A+VTS\left[\log{\left(T_{R}\right)}_{0}\right]}\right)$$

where:

- A is the intercept,
- VTS is the slope,
- η is the viscosity,
- T_R_ is the reference temperature in degrees Rankine (°R),
- (T_R_)_0_ is the initial reference temperature,
- c is a constant.

The inclusion of these formulae provides a mathematical framework for understanding viscosity-temperature dynamics, facilitating the examination of viscosity variations and transitions between different temperature points. The viscosity of the binder used in this study was measured and incorporated into the analysis to ensure accurate calculations.

#### 2.2.3. Cantabro Test

**Figure 5:**
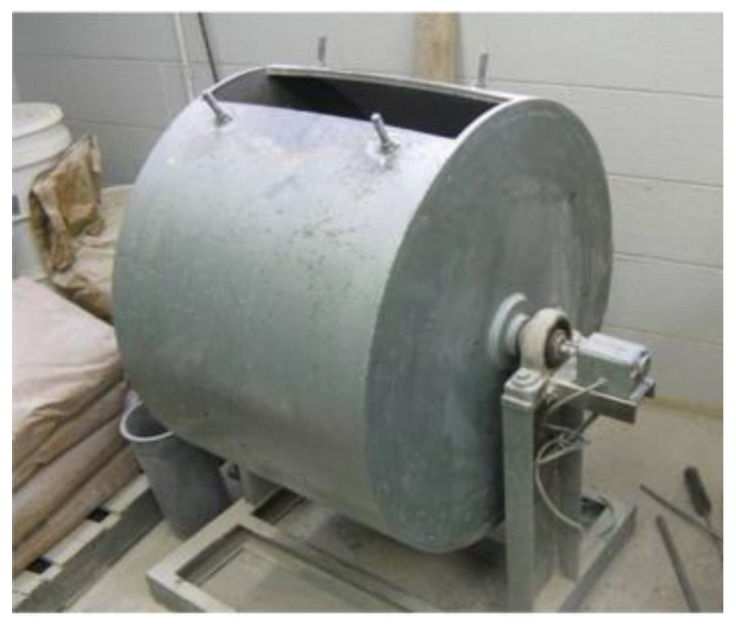
Cantabro test.

Following ASTM D6927 [45], the Cantabro experiment evaluates an asphalt concrete’s durability against fracture under continuous vehicular stress (see Figure 5). Examined in this investigation were cylindrical samples with a diameter of 100 mm and a height of 50 mm. Steel balls were dropped onto the specimen’s surface a certain number of times during the whole process. Following this preliminary stage, the sample was put in an inspection drum devoid of steel balls. The instrument ran for 300 revolutions at 28 ± 2 °C at 30–33 rpm. The sample’s mass was recorded after rotating to calculate abrasion attrition. Following every drop of order, the reduction in mass was measured, and the findings were employed to assess how long-lasting and resilient the asphalt concrete mixture was to continuous traffic loads.

#### 2.2.4. Four-Point Bending Test

**Figure 6:**
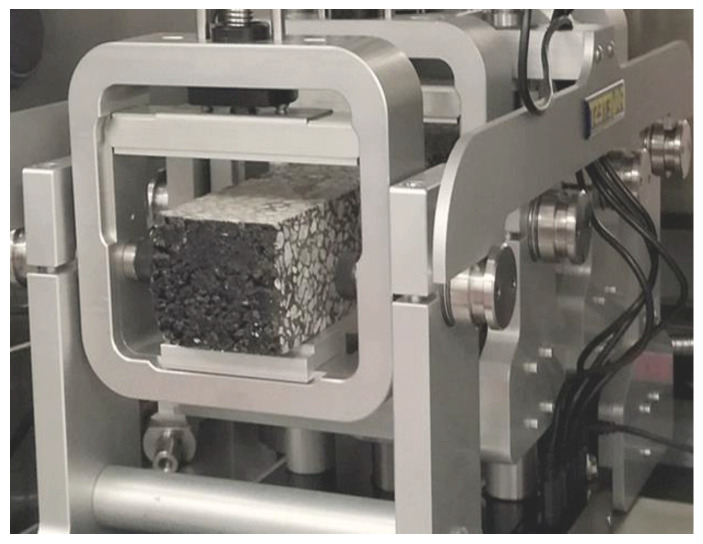
Testing apparatus of 4PB test.

The test, outlined in ASTM D6272 [46], serves as a critical method for assessing the flexural properties of the samples, offering insights into their structural behavior when subjected to bending forces (see Figure 6). During this test, a rectangular asphalt sample with specific dimensions (305 mm length, 45 mm width, 50 mm depth) is subjected to forces at two points while being supported at two others, effectively simulating real-world stresses encountered in pavement applications. Typically, specifications such as a 150 mm length of span and a 5 mm/min rate of loading are used to capture stress and displacement information. Through this process, valuable information regarding asphalt’s stiffness, flexural strength, and resistance to cracking is obtained, aiding in the evaluation of its structural performance.

#### 2.2.5. Hamburg Wheel Tracking (HWT) Test

**Figure 7:**
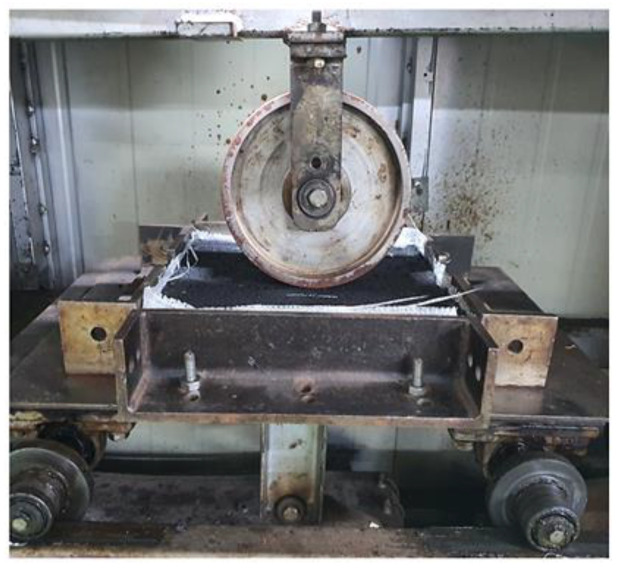
HWT results.

The Hamburg wheel tracking (HWT) test evaluates an asphalt concrete mixture’s ability to withstand rutting through modeled environmental and traffic loads. As per AASHTO T324 [47], the HWT test in this investigation was conducted with a loaded wheel measuring 700 mm in diameter (Figure 7). Before examination, the test samples, which had dimensions of roughly 150 mm in diameter by 62 mm in height, had been stored for 24 h at 60 °C. A 45 mm broad steel wheel spinning at a pace of 50 passes every minute, with the highest velocity of 340 ± 5 mm/sec at the center, applied an average stress of 700 ± 5 N to the samples throughout the experiment. Assessments of rut depth were made every 1000 cycles to assess the resilience of the combination to deformation over time. The HWT test, adhering to US State DoT guidelines, capped the permissible rut depth for hot mix asphalt (HMA) mixtures at 20 mm after 20,000 cycles.

## 3. Results

### 3.1. ITS Test

**Figure 8:**
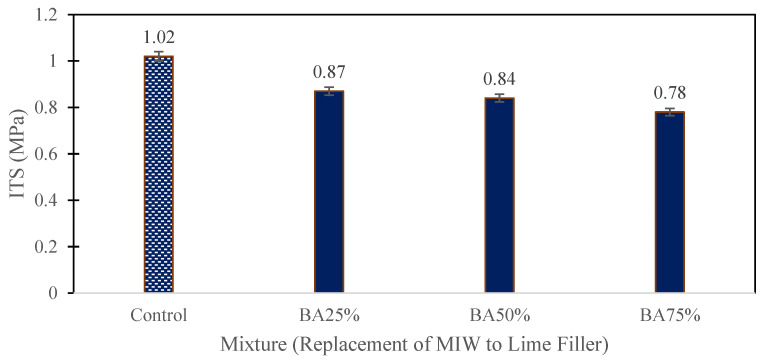
Initial ITS test results.

The ITS test results for asphalt mixtures with varying percentages of crushed bottom ash (BA) as a replacement for conventional lime filler reveal crucial insights into the mixtures’ moisture susceptibility and durability. The ITS values, expressed in megapascals (MPa), were 1.02 MPa for the control mixture, 0.87 MPa for the mixture with 25% bottom ash (BA25%), 0.84 MPa for the mixture with 50% bottom ash (BA50%), and 0.78 MPa for the mixture with 75% bottom ash (BA75%). These results showed a clear decline in tensile strength as the proportion of bottom ash increased. The control mixture’s high ITS value indicates robust performance as shown in Figure 8, while the slight reduction in the BA25% mixture’s ITS suggests that a 25% replacement level can maintain acceptable tensile strength. However, the continued decline in ITS for BA50% and BA75% mixtures highlights the adverse effects of higher bottom ash content. This trend can be attributed to the physical and chemical characteristics of bottom ash, such as its higher porosity, less uniform particle size, and different chemical composition, which may interfere with the binder’s adhesive properties and weaken the bonds within the asphalt mixture. Additionally, lime enhances binder stiffness and moisture resistance, benefits that diminish as bottom ash content increases, resulting in a less cohesive and durable mixture. Therefore, while crushed bottom ash shows potential as a filler at lower replacement levels, higher proportions compromise tensile strength and overall performance, emphasizing the need for a balanced approach to ensure effective and durable asphalt pavements, especially in moisture-prone environments.

### 3.2. Four-Point Bending Beam Test Results

**Figure 9:**
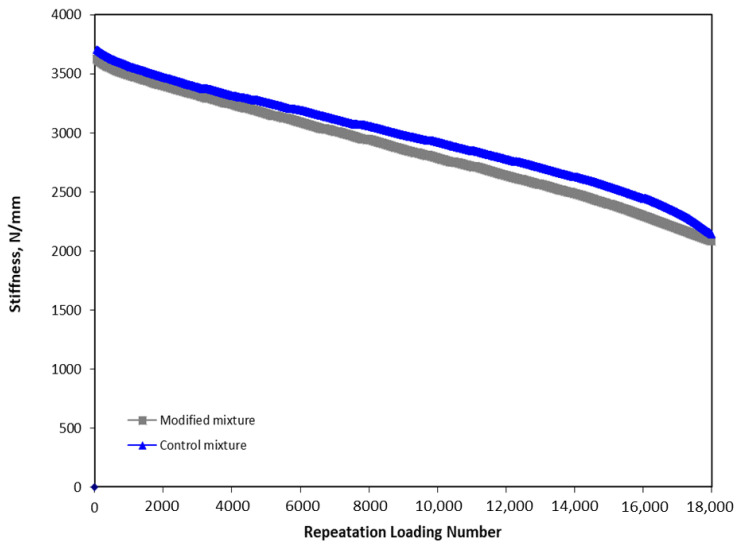
The four-point bending beam test results.

The four-point bending beam test results provide detailed insights into the stiffness behavior of asphalt mixtures containing different filler materials under repeated loading cycles as presented in Figure 9. Initially, the stiffness of all mixtures reduced sharply within the first 100 cycles, with values decreasing from 3700 N/mm to 3500 N/mm, indicating an immediate response to the applied stress. Following this initial drop, the reduction in stiffness became more gradual. Notably, during the first 5000 cycles, there was no significant difference in the stiffness reduction among the mixtures, suggesting similar initial performance regardless of the filler type.

However, beyond 6000 cycles, a distinct gap emerged. The mixture modified with bottom ash showed a higher yet stable reduction in stiffness compared to the control mixture. This behavior suggested that while the bottom ash-modified mixture experienced a greater initial drop in stiffness, it maintained a more consistent performance over extended loading cycles. In contrast, the control mixture, despite having higher stiffness initially, exhibited a more pronounced drop in stiffness after 17,000 cycles. This greater drop indicated a reduction in structural integrity and the potential for earlier failure under prolonged stress. Additionally, it is noted that at 18,000 cycles, the stiffness of the control mixture dropped to the equivalent value of the bottom ash mixture.

The mechanism underlying these observations can be attributed to the physical and chemical properties of the fillers. Crushed bottom ash, with its specific particle size and composition, may contribute to a more flexible and resilient asphalt matrix, which can absorb and dissipate stress more effectively over time. The presence of bottom ash might also enhance the binder’s ability to maintain cohesion among aggregate particles, resulting in a more stable performance under repeated loading. In contrast, the control mixture, with conventional lime filler, might exhibit higher initial stiffness but lack the same degree of flexibility, leading to a greater stiffness reduction under prolonged loading. Therefore, replacing 25% of conventional lime with crushed bottom ash appeared to provide a slight advantage by maintaining a more stable stiffness over an extended period, which can enhance the longevity and durability of the asphalt pavement.

### 3.3. Cantabro Test Results

The Cantabro test results provide valuable data on the abrasion resistance of the control and modified asphalt mixtures through weight loss measurements after specified cycles of abrasion as exhibited in Table 7. The initial and post-test weights were recorded, along with the corresponding loss percentages, to evaluate the performance of each mixture under abrasive conditions. For the control mixture, after five cycles of testing, the initial weight was 1190.2 g, and the post-test weight was 1048.9 g, resulting in a loss percentage of 11.87%. When subjected to 10 cycles, the initial weight of the control mixture was 1194.1 g, and the post-test weight was 1015.6 g, yielding a significantly higher loss percentage of 21.59%. This substantial increase in weight loss after additional cycles indicates a marked decrease in the mixture’s resistance to abrasion. In contrast, the modified mixture, which included crushed bottom ash as a partial replacement for conventional lime filler, demonstrated superior performance. After five cycles, the initial weight of the modified mixture was 1193.6 g, with a post-test weight of 1114.3 g, resulting in a much lower loss percentage of 6.66%. When tested for 10 cycles, the initial weight was 1191.8 g, and the post-test weight was 1030.7 g, resulting in a loss percentage of 13.46%.

The results clearly show that the modified mixture exhibited significantly better abrasion resistance compared to the control mixture. The lower weight loss percentages for the modified mixture, both at 5 and 10 cycles, indicate enhanced durability and cohesion among the aggregate particles. This improved performance can be attributed to the inclusion of crushed bottom ash, which may contribute to a denser and more stable asphalt matrix. The finer particle size and specific chemical composition of the bottom ash could enhance the binder’s ability to effectively coat and bind the aggregates, thereby reducing the rate of material loss under abrasive conditions. The superior performance of the modified mixture was particularly evident after 10 cycles, where the loss percentage of 13.46% was well within acceptable limits, compared to the control mixture’s 21.59%, which exceeded the typical threshold for durability. This suggests that the modified mixture with 25% crushed bottom ash filler not only resisted abrasion better initially but also maintained its structural integrity over more extended periods of stress, making it a more reliable choice for long-term pavement performance.

### 3.4. Dynamic Modulus Test Results

The dynamic modulus test results indicate that the modified asphalt mixture, incorporating 25% crushed bottom ash as a replacement for conventional lime filler, demonstrated superior performance compared to the control mixture across both high- and low-frequency stages. These stages simulated the varying conditions experienced in different weather regions, with low frequency representing hot weather regions and high frequency representing cold weather regions.

**Figure 10:**
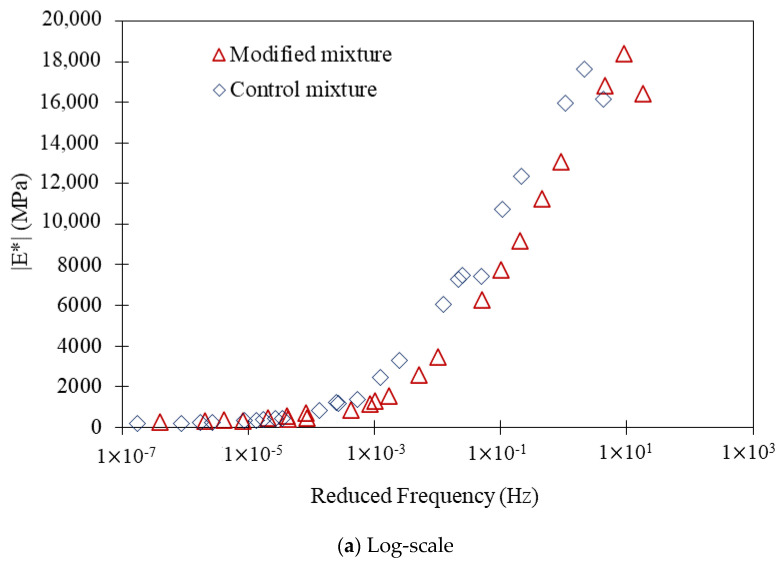

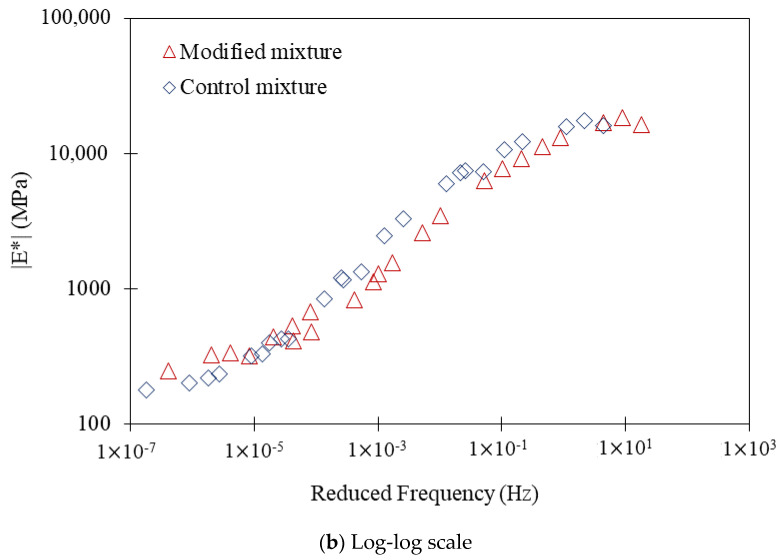
Dynamic modulus test results.

At the high-frequency stage, as shown in Figure 10a, which corresponded to the conditions in cold weather regions, the dynamic modulus of the modified mixture exceeded 18,300 MPa, whereas the control mixture exhibited an E modulus of less than 17,700 MPa. This result indicates that the modified mixture remained more rigid and resilient under rapid, repetitive loading conditions, which are common in cold environments where materials are more prone to brittleness and cracking.

Conversely, at the low-frequency stage as presented in Figure 10b, representative of hot weather conditions, the modified mixture also outperformed the control. The E modulus of the modified mixture was more than 240 MPa, while the control mixture’s E modulus was notably lower. This significant difference suggests that the modified mixture maintained better structural integrity and stiffness under the prolonged, slower loading conditions typical of high temperatures, where materials tend to soften and deform more easily.

The enhanced performance of the modified mixture can be attributed to the physical and chemical properties of the crushed bottom ash. Bottom ash typically contains a higher proportion of fine particles and a composition that includes silica, alumina, and other oxides, which contribute to improved binder-aggregate interaction. These fine particles fill the voids within the asphalt matrix more effectively, leading to a denser and more cohesive structure. This improved packing enhances the load distribution and reduces the susceptibility to deformation under stress.

Additionally, the chemical composition of bottom ash, including higher levels of silica and alumina, can enhance the stiffness and bonding characteristics of the asphalt binder. The presence of these materials likely promotes better adhesion between the binder and the aggregate particles, resulting in a mixture that can withstand higher stress levels without significant deformation. This improved bonding is particularly beneficial in hot weather conditions, where asphalt mixtures are prone to softening and rutting.

In cold weather conditions, the modified mixture’s superior performance can be linked to its ability to maintain flexibility and resist cracking. The bottom ash may contribute to a more balanced stiffness, ensuring that the mixture does not become too brittle, which is a common issue with conventional mixtures in cold climates. This balance helps the modified mixture absorb and dissipate the energy from rapid loading cycles more effectively, thereby reducing the likelihood of crack formation and propagation.

### 3.5. HWT Results

**Figure 11:**
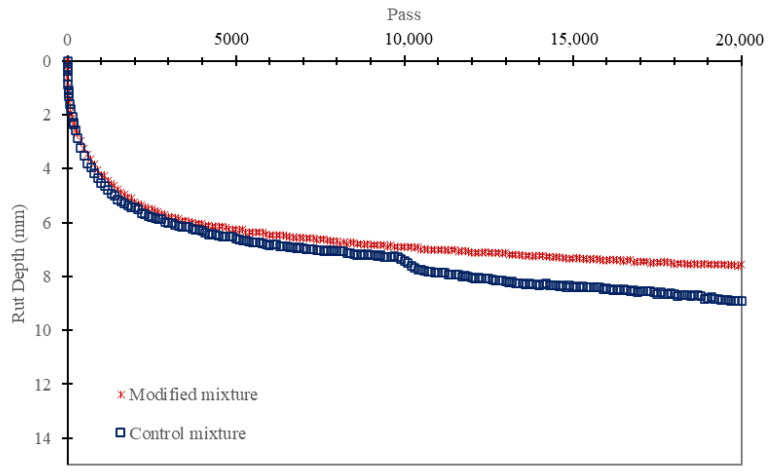
The HWT test results.

Initially, both mixtures exhibited similar behavior under the applied loads. During the first 1000 passes, the rut depth increased significantly from 0 to 4 mm (see Figure 11), primarily due to the initial compression of the material as it compacted, and air voids were reduced. This initial phase is critical as it indicates how quickly the mixture stabilized under load.

Following this initial phase, both mixtures continued to show a sharp increase in rut depth over the next 2000 cycles, with rut depths rising from 4 mm to 6 mm. This period reflects the ongoing compaction and slight densification of the mixtures as they adjusted to the repetitive loading. Between 5000 and 10,000 cycles, the rut depth for both mixtures increased more gradually, from 6 mm to 7 mm, indicating a stabilization phase where the mixtures were reaching their load-bearing capacity.

However, the gap in rutting resistance between the control and modified mixtures became evident after 8000 cycles. The control mixture experienced a stripping point of around 9800 cycles, which indicates the onset of moisture-induced damage where the asphalt binder began to lose adhesion to the aggregates, leading to a rapid increase in rut depth. In contrast, the modified mixture with 25% crushed bottom ash did not exhibit this stripping behavior, maintaining its structural integrity and resistance to moisture damage throughout the test.

At the final cycle count of 20,000, the modified mixture exhibited a rut depth of 7.56 mm, whereas the control mixture showed a greater rut depth of 8.9 mm. This significant difference underscores the enhanced rutting resistance of the modified mixture, attributed to the incorporation of crushed bottom ash.

The superior performance of the modified mixture can be explained by the physical and chemical properties of the bottom ash. Crushed bottom ash consists of fine particles that fill voids within the asphalt mixture more effectively than conventional lime, resulting in a denser and more cohesive matrix. This denser structure helps in better load distribution and reduces the potential for deformation under repeated loading. Moreover, the chemical composition of bottom ash, which includes higher levels of silica and alumina, enhances the binder-aggregate interaction, leading to stronger adhesion and less susceptibility to moisture damage. This improved adhesion prevents the stripping observed in the control mixture, thereby enhancing the overall durability and rutting resistance of the asphalt.

### 3.6. Durability Test by Using TSR

**Figure 12:**
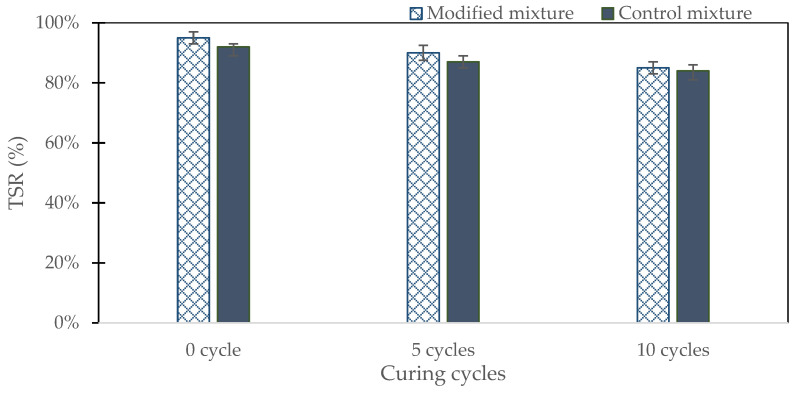
The TSR (%) test results.

The tensile strength ratio (TSR) test results after 10 wet-drying cycles provide valuable insights into the moisture susceptibility and durability of the control and modified asphalt mixtures (see Figure 12). The TSR values are indicators of how well the asphalt mixtures retain their tensile strength after being subjected to moisture damage. For the control mixture, the TSR values started at 92% at 0 cycles, dropped to 87% after 5 cycles, and further decreased to 84% after 10 cycles. This trend indicates a gradual but noticeable loss of tensile strength as the mixture underwent repeated wet-drying cycles, reflecting its susceptibility to moisture-induced damage.

In comparison, the modified mixture, which incorporated 25% crushed bottom ash as a replacement for conventional lime filler, exhibited higher TSR values at each stage. Initially, the modified mixture had a TSR of 95%, which decreased to 90% after 5 cycles and to 85% after 10 cycles. Although the modified mixture also experienced a reduction in tensile strength over time, the decline was comparable to that of the control mixture. Specifically, after 5 cycles, both mixtures showed a decrease of 5% in TSR values. However, after 10 cycles, the TSR value of the modified mixture decreased by 5%, whereas the control mixture decreased by 3% (87% vs. 84%).

The superior performance of the modified mixture can be attributed to the properties of crushed bottom ash. The finer particles with a high surface area improve packing density, reducing voids and moisture infiltration. The significant amounts of silica and alumina in bottom ash enhance bonding with the asphalt binder, improving cohesion and resistance to stripping. This stronger chemical bonding helps maintain structural integrity even when exposed to moisture. Additionally, bottom ash enhances binder adhesion to aggregate particles better than conventional lime, reducing water penetration and moisture damage. The angular shape and rough texture of bottom ash contribute to better mechanical interlocking, improving the overall stability and strength of the asphalt mixture.

## 4. Conclusions

In conclusion, this study demonstrates the potential of utilizing crushed thermal power plant bottom ash as a sustainable filler in polymer-modified asphalt concrete mixtures containing crumb rubber. Through comprehensive performance evaluations, including a range of mechanical tests, the research highlights the promising benefits of incorporating bottom ash into asphalt pavements. The following findings can be drawn from this research.

- The replacement of conventional lime filler with 25% crushed bottom ash resulted in an ITS of 0.87 MPa. Although slightly lower than the control mixture’s 1.02 MPa, the performance remained within acceptable limits, demonstrating the feasibility of bottom ash as a filler.
- The HWT test revealed that the modified mixture with 25% crushed bottom ash achieved a final rut depth of 7.56 mm after 20,000 cycles. In comparison, the control mixture had a deeper rut depth of 8.9 mm. This indicates that the modified mixture had superior rutting resistance, likely due to the better load distribution and structural integrity provided by the bottom ash.
- The adjusted mixture loosened up at a rate of 6.66% after five cycles and 13.46% following ten cycles, according to the results of the Cantabro test. This was significantly lower than the control mixture’s weight loss of 11.87% and 21.59%, respectively. The reduced abrasion loss suggests that the bottom ash enhanced the durability of the mixture by increasing its resistance to surface wear and disintegration.
- The dynamic modulus test demonstrated that the modified mixture had an E modulus of more than 18,300 MPa at low frequency and over 240 MPa at high frequency. The control mixture showed lower performance, with an E modulus of less than 17,700 MPa and around 230 MPa, respectively. The higher dynamic modulus indicates that the bottom ash-modified mixture was more resilient and maintained its stiffness better across a range of temperatures and loading frequencies.
- Tensile strength ratio (TSR) test results highlighted that the modified mixture maintained higher TSR values over 10 wet-drying cycles. Starting at 95%, the TSR dropped to 90% after 5 cycles and 85% after 10 cycles. In contrast, the control mixture started at 92%, decreasing to 87% and 84%, respectively. The improved moisture resistance can be attributed to the fine particles and chemical composition of bottom ash, which enhanced the binder-aggregate adhesion and reduced moisture infiltration.
- The stiffness of the modified mixture decreased sharply in the first 100 cycles, similar to the control mixture, but then remained stable beyond 6000 cycles. The control mixture, however, exhibited a significant drop in stiffness after 17,000 cycles. This indicates that the modified mixture with bottom ash was more stable and durable under repeated loading, providing better long-term performance.
- Utilizing crushed bottom ash as a filler in asphalt mixtures not only improves performance but also offers significant environmental and economic advantages. It provides a sustainable solution by recycling industrial waste, reducing the dependency on conventional lime, and potentially lowering material costs. This contributes to more eco-friendly pavement construction practices and supports waste management efforts.

In summary, replacing 25% of conventional lime filler with crushed bottom ash in polymer-modified asphalt concrete mixtures containing crumb rubber significantly enhanced the mechanical properties, durability, and environmental sustainability of the pavement. The modified mixture exhibited superior rutting resistance, abrasion resistance, dynamic modulus, and moisture resistance, making it a highly viable alternative for high-performance and long-lasting asphalt pavements.

## Figures and Tables

**Table 1:** Sieve analysis of aggregate.

Sieve Size (mm)	Percentage Passing
19.0	100
12.5	90–100
9.5	70–90
4.75	35–55
2.36	23–35
1.18	15–25
0.600	10–20
0.300	5–15
0.150	2–10
0.075	0–5

**Table 2:** Aggregate properties.

Property	Value
Specific Gravity	2.65
Bulk Density	1.6 g/cm^3^
Water Absorption	1.0%
Los Angeles Abrasion	20%
Aggregate Crushing Value	25%
Flakiness Index	10%

**Table 3:** Summary of bottom ash properties.

Property	Value
Particle Size Range	0.1–100 μm
Specific Surface Area	25 m^2^/g
Total Porosity	40%
Density	2.7 g/cm^3^
SiO_2_	50%
Al_2_O_3_	28%
Fe_2_O_3_	12%
CaO	6%
MgO	2%
SO_3_	2%

**Table 4:** Characteristics of SBS polymer.

Characteristic	Measurement
Tensile Strength	5.0 MPa
Elongation at Break	700%
Softening Point	75–95 °C
Penetration Index	40
Complex Shear Modulus (G*)	1.85 kPa at 64 °C
Phase Angle (δ)	75° at 64 °C

**Table 5:** Recycled crumb powder’s properties.

Property	Value
Mesh Size	20–40 mesh
Density	0.5–0.8 g/cm^3^
Moisture content	≤1%
Rubber content	≥90%
Elongation at Break	200–400%
Specific Gravity	1.12–1.14
Softening Point	65–85 °C

**Table 6:** Properties of HMA used in this research.

Properties	Value
Penetration (1/10 mm) 25 °C [34]	70.2 (1/10 mm)
Softening Point (°C) [35]	74.5 °C
Ductility at 5 °C (cm/min) [36]	110 cm/min
Mass Loss (%) [37]	0.038%
G*/sinδ at 64 °C (Original) [38]	1.85 kPa
G* × sinδ at 64 °C (after PAV) [39]	1520 kPa
Stiffness at −22 °C [40]	190 MPa
m-value at −22 °C [40]	0.35

**Table 7:** Summary of Cantabro test results.

	Initial Weight (g)	Weight Post-Test (g)	Loss Percentage (within 20% Based on Drainage)
Control mixture: 5 cycles	1190.2	1048.9	11.87%
Control mixture: 10 cycles	1194.1	1015.6	21.59%
Modified mixture: 5 cycles	1193.6	1114.3	6.66%
Modified mixture: 10 cycles	1191.8	1030.7	13.46%

## Data Availability

The raw data supporting the conclusions of this article will be made available by authors on request.
